# XPS Characterization of TiO_2_ Nanotubes Growth on the Surface of the Ti15Zr15Mo Alloy for Biomedical Applications

**DOI:** 10.3390/jfb14070353

**Published:** 2023-07-05

**Authors:** Reginaldo Toshihiro Konatu, Danielle Duque Domingues, Rodrigo França, Ana Paula Rosifini Alves

**Affiliations:** 1 School of Engineering and Sciences, Guaratingueta Campus, São Paulo State University (UNESP), Guaratinguetá 12516-410, Brazil; reginaldo.konatu@unesp.br; 2 School of Engineering, Ilha Solteira Campus, São Paulo State University (UNESP), São Paulo 15385-000, Brazil; danielle.d.domingues@unesp.br; 3 Department of Restorative Dentistry, University of Manitoba, Winnipeg, MB R3E0W2, Canada

**Keywords:** titanium alloy, anodization, TiO_2_ nanotubes, XPS

## Abstract

Ti15Zr15Mo (TMZ alloy) has been studied in recent years for biomedical applications, mainly due to phase beta formation. From the surface modification, it is possible to associate the volume and surface properties with a better biomedical response. This study aimed to evaluate the possibility of using anodization to obtain TiO_2_ nanotubes due to the presence of valve-type metal (Zr) in their composition. X-ray photoelectron spectroscopy (XPS) was performed to determine the surface chemical composition in both after-processing conditions (passive layer) and after-processing plus anodization (TiO_2_ nanotube growth). The anodization resulted in nanotubes with diameters and thicknesses of 126 ± 35 and 1294 ± 193 nm, respectively, and predominated anatase phase. Compared to the passive layer of titanium, which is less than ~10 nm, the oxide layer formed was continuous and thicker. High-resolution spectra revealed that the oxide layer of the element alloys contained different oxidation states. The major phase in all depths for the nanotube samples was TiO2. While the stable form of each oxide was found to predominate on the surface, the inner part of the oxide layer consisted of suboxides and metallic forms. This composition included different oxidation states of the substrate elements Ti, Zr, and Mo.

## 1. Introduction

In recent years, titanium and its alloys have been used as biomaterials, mainly to manufacture dental implants, due to their excellent mechanical properties, low specific weight, high corrosion resistance, and biocompatibility [1]. Over the past few years, titanium-based alloys with different compositions, such as Ti7.5Mo [1,2], Ti5Zr, Ti10Zr, Ti35Nb5Zr, and Ti35Nb10Zr [3], have been studied for biomedical applications. Ti6Al4V is often the material chosen for this application, and although this alloy presents good biocompatibility and corrosion resistance, when in contact with cells or human fluids, aluminum and vanadium may be related to adverse reactions within the body [3,4,5,6,7,8,9,10,11].

In this sense, novel Ti-based alloys are being developed vanadium/aluminum-free to improve properties, promote biofunctionalization, and reduce the differences between bone and mechanical implant properties, decreasing complications that can generate [12], for example, the stress shielding effect [4,5,13,14,15]. Among these alloys, studies have focused on beta (β) titanium alloys because they exhibit superior mechanical properties suitable for biomedical applications, such as lower elastic modulus, high mechanical strength, good conformability, and high ductility [4,16]. The effect of the substitutional elements on the microstructure and mechanical behavior of the ternary alloy system Ti15Zr15Mo (TZM) was evaluated by Correa et al. (2018) [17]. The authors concluded that TZM alloy presented only equiaxial grain formation of β phase and the best potential for a possible biomedical application due to an adequate combination of β-phase and mechanical properties.

Surface properties are crucial in the alloy’s performance [18,19]. Properties such as surface composition, surface roughness (micro and nanoscale), and surface energy can accelerate the time of cell adhesion and proliferation. This decrease in healing time can reduce the chances of micromotions and guarantee implantation success [3].

In recent years, our group has been studying the anodization of the new β titanium alloys for biomedical applications with the goal of better bulk and surface properties [1,2,20,21,22,23]. The morphology of the nanotubes is influenced by anodization parameters such as voltage, electrolyte, and time. Also, the diameter and depth of the nanotubes can be varied by changing the alloy composition. For example, the study of the binary system TiMo showed that the molybdenum content influences the morphology of the nanotubes in the anodization process due to the constituents of the oxide layer [24].

The anodization of both Ti15Zr and Ti15Mo binary systems has been successfully performed in our previous studies. Rangel et al. [22] studied nanotube growth on the Ti15Mo surface and obtained nanotubes with 65 nm and 500 nm thickness while values of 80.6 nm of diameter and 1370 nm of thickness were observed in Ti15Zr alloy with improvement in the cell response [25]. The MAO (Micro Arc Oxidation) is another technique used to change the surface due to the presence of valve-type materials such as Zr. It was employed for surface modification of the Ti15Zr10Mo and Ti15Zr15Mo1Ag [17,26]. These studies used MAO treatment to obtain bioactive surfaces in the alloys developed to be applied as osseointegrated implants. Correa et al. [17] found exhibited porous MAO films with pore size and thickness of <4.5 μm and 15 μm, respectively, and low crystallinity, composed of inner and outer amorphous layers and an intermediary nanocrystalline region of mixed anatase and rutile phases. Immersion in Hanks’ solution for 7 days showed that MAO-treated samples presented noticeable improvement in the bioactive response, and biological tests demonstrated that Ti-15Zr-xMo alloys and MAO-treated surfaces were biocompatible. Torrento et al. [26] paper concluded that MAO treatment also produced a porous oxide layer, and phase composition and microstructure were composed by the β phase as equiaxial grains. The combination of bulk alloying and surface treatment made the samples good choices for biomedical implants. These results suggest that the combined effect of textured surfaces and surface enrichment with Ca and P is a very promising approach to promoting bone formation. 

Our goal in this work was to characterize the surface layer of the Ti15Zr15Mo alloy and thus show the viability of using anodization for the surface modification, as it is a more economically viable technique when compared to MAO.

## 2. Materials and Methods

The Ti15Zr15Mo alloy processing was based on our previous studies for the binary alloys Ti15Zr and Ti15Mo [22,25]. Briefly, titanium (grade 2), zirconium (99.9%), and molybdenum (99.9%) were weighed according to the alloy composition and melted in arc-melting-furnace in an argon atmosphere at least ten times which allowed the homogeneity of the alloy. Anodization was carried out on the surface of the discs (10 mm diameter and 3 mm height). Two parameters were evaluated: voltage and time. 

Samples were anodized in an electrolyte prepared by mixing water, glycerol (50 V%), and ammonium fluoride (NH_4_F 2.7 g/L). This experiment used two different voltages, 20 and 30 V, with a voltage rate of 2 V/min, performing for 6 and 24 h. After the anodization, all samples were annealed at 450 °C for 1 h to crystalline phase stabilize. Thus, four groups were evaluated: 20 V/6 h, 30 V/6 h, 20 V/24 h, and 30 V/24 h. 

Processed alloy microstructures were characterized by light microscopy and X-ray diffraction (XRD). The samples for microscopy were polished and etched with Kroll’s reagent to be analyzed with a light microscope (Epiphot 1000, Nikon, Japan). The samples for XRD were polished, anodized for 24 h and 30 V, and performed in an X-ray diffractometer (D8 Advance Eco, Bruker, MA, USA) using radiation Cu Κα (λ = 1.54 Å), 2θ = 20°–90°. 

Surface characterization was performed on the alloys after anodization to choose one group to be further analyzed according to the crystalline chemical characterization. The TiO_2_ nanotubes surface characterization was carried out by a scanning electron microscope (SEM/FEG-Magellan 400L, FEI, OR, USA), and the criteria to choose it was the higher nanotube diameter, exploring the possibilities of using nanotubes as a container for other purposes. Nanotube diameter was measured with ImageJ software [27], and all images were calibrated using a scale bar and converted to 8-bit. The threshold identifies the inner diameter and analyzes particles to measure the inner diameter. The studying particle setup was from size 200 to infinity, effectively removing noise and circularity from 0.75 to 1.00. Circularity filters result from a single particle to a particle cluster. The overall measurement results were summarized in a statistics table, obtaining a normal distribution. Minitab was used to plot a boxplot of nanotube diameter and Pareto’s chart for standardized effects. 

X-ray photoelectron spectroscopy (XPS) (Axis Ultra, Kratos, UK) was performed with monochromatic Al Κα radiation (1486.6 eV), a voltage of 15 kV, and a neutralizer. This characterization was carried out with a depth profile with argon ablation and at three different times: 0 (without ablation), 75, and 750 s. In addition, the survey spectrum and a high-resolution spectrum of carbon, oxygen, and titanium were analyzed. The XPS results were analyzed using CasaXPS (Casa, UK, Software version 2.3.23PR1.0). The following steps were taken to ensure accurate analysis: Firstly, the energy calibration was fixed using the carbon C 1s peak at 285 eV. Secondly, background subtraction was performed using a Shirley background removal method. Lastly, peak fitting was carried out using a Lorentzian–Gaussian function with a ratio of 40:60. These steps ensured precise and reliable analysis of the XPS results.

## 3. Results and Discussion

**Figure 1:**
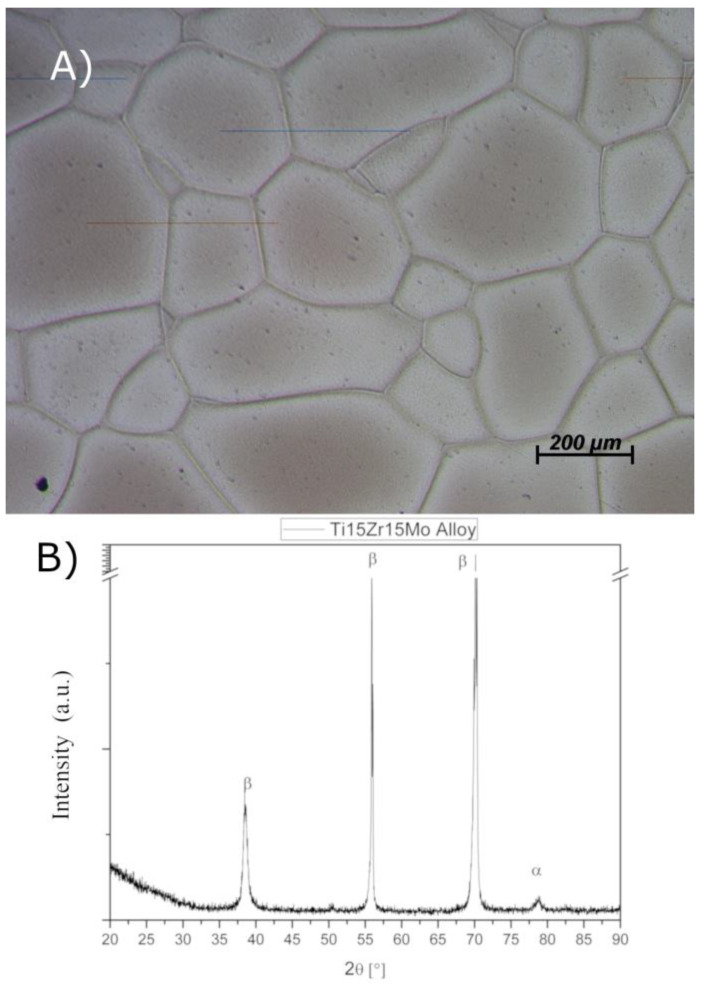
(**A**) Micrography of the Ti15Zr15Mo alloy after etching with Kroll’s reagent. (**B**) X-ray diffraction of the Ti15Zr15Mo alloy without surface treatment (lower) and after anodization with TiO_2_ nanotubes (identified by the presence of TiO_2_ anatase peaks). JCPDS card No. 01-078-07533.

The composition of the alloy was prepared by weighing pure metal in the ratio of the alloy: 15 wt% Zr, 15 wt% Mo, and Ti balance. Melting and swaging processes of this alloy resulted in a predominant β-titanium alloy. Therefore, it is possible to see that Figure 1A shows equiaxial grains between 100 and 550 µm of β-phase, which is the predominant phase of this alloy.

In Figure 1B, X-ray diffractograms of the titanium alloy show β-phase predominance, confirmed by using a Ti β-phase pattern (PDF 44-1288) and diffraction planes (110), (200), (220), and (211). In addition, the β peaks found in the sample are associated with peaks observed in titanium alloy reported by other authors [28,29].

The grain size of titanium alloy processed as described in this paper is coherent with that found in the literature, with an average diameter of 100–250 µm. Studies from Cardoso et al. (2014) [28], Qin et al. (2014) [10], and Zhao et al. (2012) [29] processed the alloy by hot roll, while Nnamchi et al. (2016) [30] processed it by hot swage, all resulting in grain size in the same order of magnitude. Grain size differences are attributed to the reduction rate of the alloy. Equiaxial grain and this size of grain are characteristic of β titanium alloy. Despite the authors working with different titanium alloys, the rate of β stabilizer is higher than 10 wt%, resulting in a β titanium alloy; on the other hand, when it is near 10 wt%, it was found α” + β titanium alloy. Molybdenum is a strong β-stabilizer [4,25], so the results are according to the literature. Beta titanium alloys show promise for biomedical applications due to their low elastic modulus when compared to alfa (α) and alfa+beta (α+β) alloys or stainless [23,31].

There is a critical concentration of β elements alloy in binary titanium that retain β-phase after quenching to room temperature. Molybdenum critical concentration is 10 wt% [32]. Zirconium is a neutral element and does not strongly influence β-transus; however, this element may influence mechanical properties [33]. The titanium alloy studied in this paper has 15 wt% Mo and 15 wt% Zr. X-ray diffractions confirm the presence of β-phase as in the papers of Rangel et al. [22] and Correa et al. [16], being able to affirm that titanium alloy with a concentration of 15 wt% of molybdenum stabilized only β phase and equiaxial grains. 

**Figure 2:**
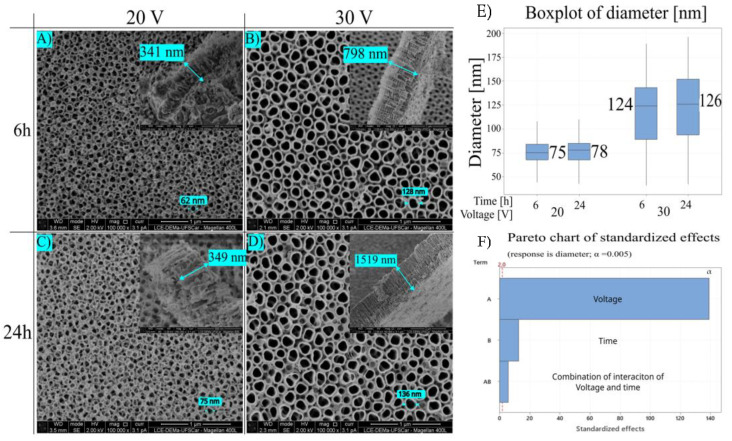
Ti15Zr15Mo alloy anodized by (**A**) 20 V and 6 h; (**B**) 30 V and 6 h; (**C**) 20 V and 24 h; (**D**) 30 V and 24 h. Scale bar of thickness image of Figure (**A**) 500 nm; (**B**) 1 µm; (**C**) 500 nm; (**D**) 2 µm. (**E**) Boxplot of nanotube diameter comparing each condition (centerline of boxplot is the median value of group). (**F**) Pareto Chart of standardized effects comparing A—voltage, B—time, AB—interaction between voltage and time.

In the present paper, TiO_2_ nanotubes were grown on Ti15Zr15Mo alloy by anodization oxidation in an organic electrolyte. The electrolyte comprised glycerol, water (50 vol%), and ammonium fluoride (NH_4_F, 2.7 g/L). Anodization was performed with different voltage parameters (20 and 30 V) and times (6 and 24 h). All groups used in this experiment resulted in a nanotube layer. Surface images of samples are shown in Figure 2 A–D, and the upper right image presents a thickness layer SEM.

Escada et al. (2013) reported nanotube layer growth over Ti7.5Mo using a similar electrolyte with 10 vol% of water and 2.5 g/L of NH_4_F nanotube with 80 nm diameter [1], while Chaves et al. [1] obtained nanotubes growth over Ti15Mo alloy using the same parameters as Escada, resulting in a nanotube diameter of 65 nm [21]. Rangel et al. [22] also reported a diameter of 65 nm using similar parameters as Escada and Chaves, but with a different time of 6 h against 24 h used by Chaves and Escada, resulting in a difference in nanotube morphology [22]. The nanotube layer influences substrate (titanium alloy), electrolyte, and different anodization parameters [34]. 

Figure 2E shows a boxplot of the nanotubes’ diameter, and the range of values for each group may be seen. The box indicates that 50% of the results are in that area, and the central bar indicates this data median. These distributions suggest a significant difference between the groups with different voltages and a slight difference compared to the process’s time. For example, the distribution contains diameter values for 30 V, similar to the 20 V group. 

There is a region below the box that can represent a low diameter, and these structures can be attributed, in major part, to a void between the nanotubes. These voids are formed during the dissolution of the oxide layer and the formation of the nanotubes. The presence of this void can differentiate nanoporous and nanotubes [28].

Figure 2F represents a Pareto chart of voltage and time-standardized effects in diameter of TiO_2_ nanotube growth on Ti15Zr15Mo alloy. This result outlines that voltage, process time, and their combination statistically affect the nanotube’s diameter. Voltage is directly related to diameter growth. 

Roy et al. [35] concluded that nanotube diameter has a linear relation with the potential applied in the process. Yasuda and Schmuki [36] discovered that anodization potential is a factor in controlling nanotubes’ aspect ratio, thickness, and diameter. Several works reported voltage as an essential factor controlling nanotube diameters [3,36,37,38]. 

From Table 1, it can be seen that the mean diameter was measured using an SEM micrograph. In this paper, there is a significant difference in diameter and thickness when comparing groups of 20 V and 30 V. However, when we vary the anodization time, 6 and 24 h, there is a slight difference in the diameter. The results from Table 1 show that the highest diameter was 121.9 nm and the highest thickness was 1294 nm, obtained at 30 V and 24 h.

**Figure 3:**
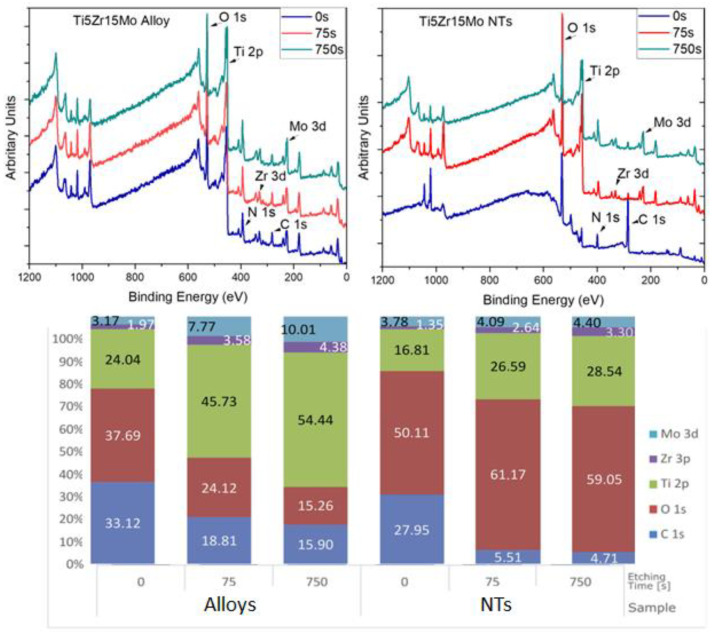
XPS survey of alloy and samples of the alloy after anodization on the surface 0 s, 75 s, and 750 s of argon etching. Diagram of the percentage of concentration of elements (C, O, Ti, Zr, Mo) present on the sample’s surface (etching time of 0) and with different times of argon.

Figure 3 displays the XPS chemical analysis (survey spectra) of elements on the surface. Depth profiling by argon etching revealed the inner layer of these surfaces, and it was preceded by 75 s and 750 s (corresponds to 10 nm and 100 nm, respectively). The surface and layers are composed of carbon (C), oxygen (O), titanium (Ti), zirconium (Zr), and molybdenum (Mo). 

From the results, we can confirm the presence of carbon on the surface of both samples, and it was used to calibrate them with carbon 285 eV. This adventitious carbon was attributed to contamination or sample manipulation. Carbon represents approximately 30% in both samples, reducing this percentage after argon ablation to 15.9% and 4.7% (sample alloy and NTs, respectively). 

Papers in the literature have found fluorine on the interface of metal/oxide of nanotubes after anodization [35,39]. The presence of fluoride ions helps to determine the oxide layer on the amorphous TiO_2_ nanotube layer. Fluorine is one of the most common elements in electrolytes for anodization, and its presence is attributed to a residual electrolyte on the surface, or it may be kept inside the crystalline structure of TiO_2_. In these studies, the presence of fluorine on the surface was reported before heat treatment (annealing), and after heating treatment, fluorine was removed from the surface due to evaporation temperature [36]. This explains the absence of fluorine on the nanotube sample’s surface, resulting in our study. 

Oxygen is attributed to the organic compounds and oxide layer. A significant percentage of oxygen is related to the metal oxide layer. There is a difference between the oxygen content on the alloy and the nanotube samples. It can be explained by the oxide layer thickness present on both samples because on Ti15Zr15Mo alloy, there is only a titanium passive layer, and on Ti15Zr15Mo, after anodization, there is a nanotube oxide layer. This layer is thicker than the passivation one.

Metals such as titanium, zirconium, and molybdenum are attributed to the substrate and to the composition of the oxide layer. Samples of Ti15Zr15Mo alloy show a higher oxygen concentration in the first analysis without argon etching. In contrast, the metal percentage is more than 50% of atoms after etching, and the other part is formed by carbon and oxygen. This can indicate the reduction in the passive layer (TiO_2_). After the second argon etching, oxygen and carbon continue to reduce, whereas the metals concentration of titanium, zirconium, and molybdenum increase. Results for Ti15Zr15Mo after anodization samples show a higher concentration of oxygen and a reduction in carbon rate and the other metals. Argon etching of 75 and 750 s on the surface reduces carbon but keeps the rate of oxygen and metals stable. Therefore, we can conclude that the oxide layer is not entirely removed by argon etching of 750 s. This result corroborates with data in Table 1; in all groups, the samples have more than 300 nm of thickness.

The rate of titanium and oxygen (1:2) determines the presence of an oxide layer on the surface. Ti15Zr15Mo alloy samples showed a rate near this proportion on the first analysis, and the metal concentration increased over the oxygen. As a result, the maximum atomic percentages of titanium oxide were 12% (75 s etching) and 7% (750 s etching). On the other hand, on Ti15Zr15Mo, after anodization samples, there is an amount of oxygen more than twice the metals concentration, which can form the stoichiometric forms of titanium oxide (TiO_2_), zirconium oxide (ZrO_2_), and molybdenum oxide (MoO_2_ and MoO_3_). 

The high-resolution XPS analysis of elements carbon (C 1s), oxygen (O 1s), titanium (Ti 2p), zirconium (Zr 3d), and molybdenum (Mo 3d), the oxidation state, and/or elements bonded are shown in Table 2. Carbon (285 eV) was used as a reference for calibration. High-resolution spectra of carbon show peaks predominantly of carbon bonds with carbon, hydrogen, or oxygen (C-C, C-H, C-O) at 285 eV. Carbon and carbon bonds with oxygen are C-O at 286.16 and O-C=O at 288.72. As the carbon source was from contamination, it is challenging to analyze a sample without carbon carbonates that can be deposited on the surface of TiO_2_ in a few seconds [40].

**Figure 4:**
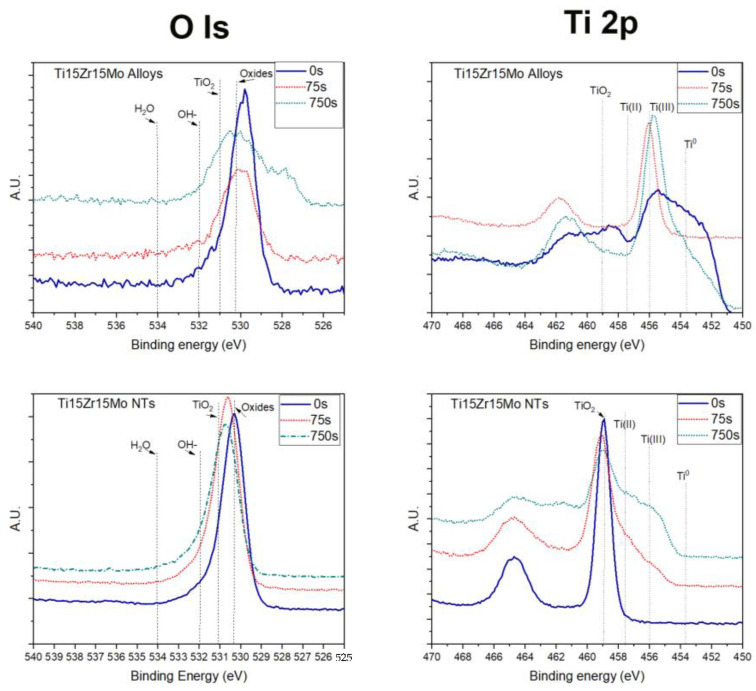
XPS analysis of the Ti15Zr15Mo alloy and the alloy after anodization. Survey spectrum and a high-resolution spectrum of oxygen and titanium in three different depths of analysis.

Deconvolution peaks of oxygen (O 1s) were attributed to metal oxides (TiYO_x_; ZrO_x_; MoOx at 530.6 eV and TiO_2_ at 531 eV) and an organic group ( 532.33 eV and 534.8 eV), as shown in Figure 4. 

The results from the titanium alloy sample presented an intense peak attributed to metal oxides on the surface, and after the first argon ablation, the shape of the oxygen peak changed. In addition, data show metal oxides reduction and increasing titanium dioxide (TiO_2_). On the other hand, for the alloy samples after anodization, there are predominantly titanium dioxide (TiO_2_), and the shape of the peak is related to the passive layer covering titanium alloy. These findings corroborate with SEM results of a nanotube layer whose thickness was greater than 1249.2 nm for the sample with anodization parameters 30 V and 24 h. 

High-resolution spectra of titanium (Ti 2p) of the samples showed double peaks of titanium (Ti 2p3/2 and Ti 2p1/2) located at 458.7 eV and 464.4 eV (split orbital = 5.7 eV); these peaks were attributed to Ti^+4^ oxidation state (TiO_2_), the stable oxide state of titanium. Also, deconvolution of the peak Ti 2p3/2 revealed other intermediate oxidation states of titanium: TiO (Ti^+2^) and Ti_2_O_3_ (Ti^+3^) located at 455.73 and 457.41 eV, respectively. The peak of Ti 2p3/2 at 453.53 eV is attributed to the titanium metallic state (Ti^0^) and their second peak (Ti 2p1/2) at 459.63 eV; there is a split orbital of 6.1 eV for the metallic state of titanium.

Figure 4 shows Ti15Zr15Mo alloy high-resolution spectra at different layers of analysis (0, 75, and 750 s of argon etching) for titanium alloy and TiO_2_ nanotube samples. It is possible to see the evolution of their composition by comparing the shape of each layer’s curve after removing some atoms’ nanometers, according to the surface thickness. From the results of titanium alloy, we can observe a shift to the low binding energy and a more defined peak of metallic state. On the other hand, samples of the alloy after anodization stay in the same position, increasing the base of the curve by the presence of a new state of oxidation of titanium. 

**Figure 5:**
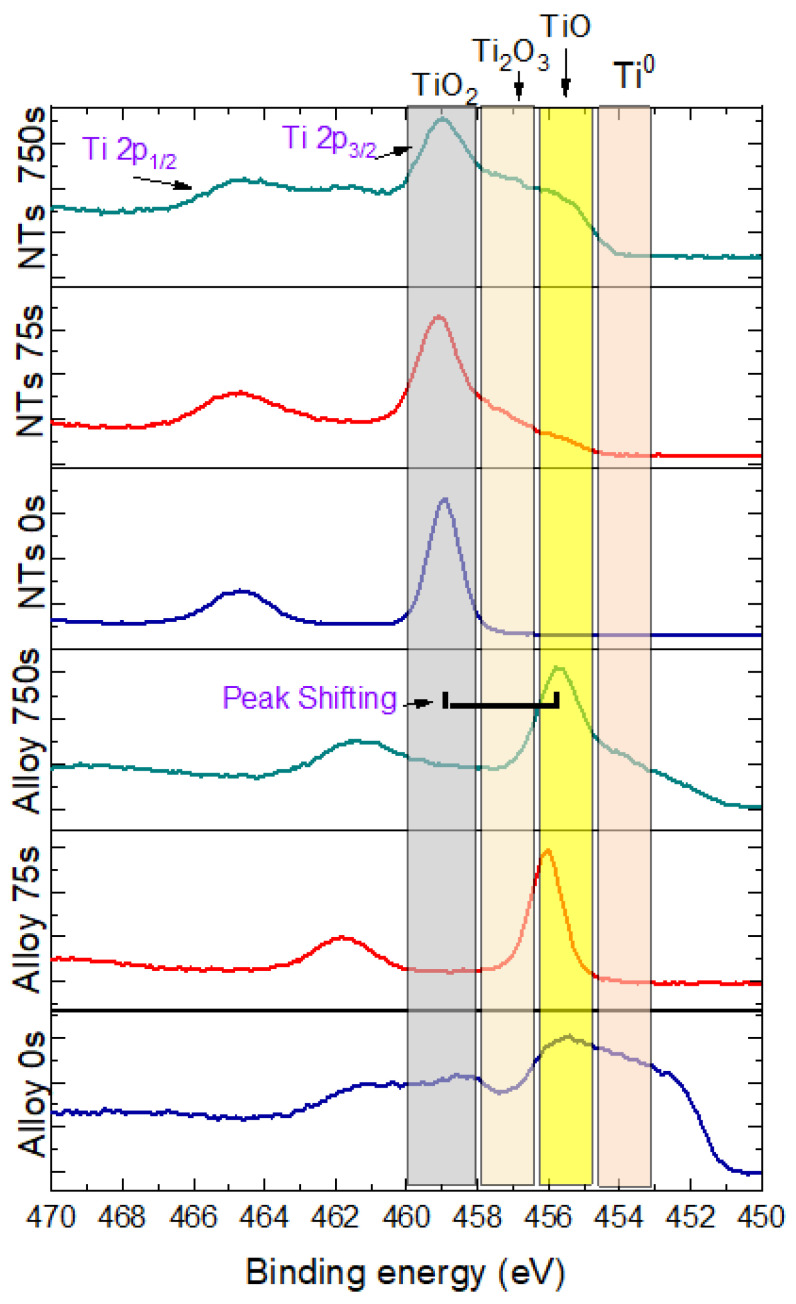
XPS analysis of NT’s Ti15Zr15Mo alloy and nanotube. A high-resolution spectrum of titanium and deconvolution compares two depths (0 s, 75s, and 750 s of argon etching).

Figure 5 shows the deconvolution of titanium (Ti 2p) in different titanium oxidation states in two layers of analysis with 0 and 750 s etching for titanium alloy and nanotube samples. The figure shows the presence of TiO, TiO_2_, and Ti_2_O_3_ at 0s etching, which are attributed to the passivation layer of titanium alloys. After 75 s etching, this layer was removed, evidencing the substrate (metallic state—Ti^0^). The results for the samples of the alloy after anodization, high-resolution spectra showed the predominant presence of TiO_2_ in all layers analyzed, and after the first etching, there are low binding energy peaks. These peaks were attributed to titanium oxides and suboxides (TiO_2_, TiO, and Ti_2_O_3_), but there was no evidence of metallic titanium (Ti^0^). The oxide layer’s presence shows that the nanotube layer’s thickness is more extensive than 100 nm.

The XPS spectrum of zirconium was deconvoluted into three groups of peaks, paired with split spin-orbital components of 2.43 eV. The groups found are Zr 3d5/2 at the position of 182.90 eV and was attributed to ZrO_2_, Zr 3d5/2 was attributed to ZrO, and Zr 3d5/2 was attributed to metallic zirconium (Zr^0^). It was found in results for “no etching” alloy samples the predominance of zirconium oxides (ZrO, ZrO_2_), while after 75 s etching, most of the stable oxide was removed, reducing the rate of zirconium dioxide (ZrO_2_) and increasing zirconium metallic. After that 750 s etching, zirconium metallic was dominant compared to the other one, and it is possible to conclude that part of the surface was revealed. The data obtained for alloy samples after anodization present peaks attributed to a zirconium dioxide (ZrO_2_–Zr 3d5/2) peak at 182.9 eV and another peak at 182.29 eV attributed to zirconium oxide (ZrO). There was a mix of these two oxide states independently of the oxide layer.

Molybdenum deconvolution presents three peaks referent to metal (Mo^0^), MoO_2_, and MoO_3_ at positions of 226.91 eV, 228.91 eV, and 234.34 eV, respectively. Mo 3d5/2 and 3d3/2 have a splitting of 3.15 eV. Titanium alloy samples showed a different behavior for molybdenum. In contrast to other metals described previously in this work, the peak related to MoO_2_ was the highest, and Mo metallic is the second in all layers of analysis. This can reflect the possibility of metallic molybdenum presence on the oxide layer. In the alloy samples after anodization, high-resolution spectra were deconvoluted in three peaks: 227.91 eV attributed to metallic molybdenum, 228.91 eV attributed to MoO_2_, and 234.32 eV attributed to MoO_3_. At the 0 s layer (“no etching”), there were no signals of metallic state but were predominantly composed of MoO_3_. After the etching of 75 s, metallic molybdenum and other forms of molybdenum oxide (MoO_2_, MoO_3_, Mo_2_O_5_) were noticed. At the 750 s layer, there was more Mo in the metallic state, and MoO_2_ was remarked.

Metal elements, such as titanium, zirconium, and molybdenum (Ti, Zr, and Mo), are predominantly in a stable oxide state (TiO_2_, ZrO_2,_ and MoO_3_) without argon ablation (0 s). However, after the first ablation, there is a dislocated peak to the suboxide state, indicating there is a suboxide form of metals (e.g., TiO, Ti_2_O_3_, ZnO) in the nanotubes composition, or it may result from removing an atom of oxygen by a collision of argon, changing the structure of the oxide.

When comparing the XPS results from depth profiling of Ti15Zr15Mo alloy samples and the alloy with nanotubes, we can observe differences in composition, confirming the distinction between oxide thickness. The oxide layer thickness of titanium alloy and alloy samples after anodization diverges according to surface composition between the analyses of different layers (“no etching”, 75 s, and 750 s etching). The first relation analyzed compares titanium and oxygen rate on different layers of the samples. The results thus obtained evidence of the presence of different titanium oxidation.

Titanium oxide titanium dioxide (TiO_2_) has a Ti/O ratio of 1:2 to exist. More oxygen than titanium can represent the presence of other metal oxides, e.g., zirconium dioxide and molybdenum dioxide (ZrO_2_ and MoO_2_). It can be from different sources, e.g., organic contaminants. When there is more titanium than oxygen, this can represent the existence of other oxidation states, such as suboxides or metallic states. The analysis of a metallic state percentage higher than the other titanium oxidation states indicates the presence of a metallic surface exposed due to argon ablation, removing this metallic oxide from the surface.

Titanium alloy samples showed a Ti:O ratio of 1:2 only on surface analysis, which is the “no etching” condition (0 s etching). However, after 75 s etching, it can be seen that more titanium than oxygen and the concentration of metallic titanium increases. Due to these facts, this group’s passive layer corresponds to less than 10 nm of thickness. On the other hand, samples of the alloy after anodization showed a constant Ti:O ratio in all three-time conditions: 0 s, 75 s, and 750 s etching. Therefore, the TiO_2_ nanotube layer was thicker than 100 nm (750 s etching). 

The results from alloy samples after anodization depth profiling shows another tendency related to the oxidation state of titanium changes at different layers. On the surface, the oxidation state predominant is Ti^+4^; after argon etching of 75 s, the predominant oxidation state of titanium is Ti^+3^; and the last etching titanium oxidation state is Ti^+2^. The deeper or closer to the substrate/oxide interface, the metallic species with less oxygen linked are predominant due to the restriction of oxygen. The oxygen moves from the surface of the oxide layer by diffusion to the metal/oxide interface. When oxygen atoms find the metal interface, they form an oxide that grows from inside to outside.

Zirconium and molybdenum are alloy elements present in titanium alloy. The presence of Zr and Mo on the oxide layer of the titanium alloy sample and nanotube sample originates from the substrate. These elements can move from the alloy to the oxide as part of oxidation. The presence of these elements on Zr and Mo is attributed to metal oxide and the metallic state of the element (presence in the substrate). Molybdenum has the metallic form mixed with the oxide that makes up the nanotubes, a fact that can occur due to the growth of titanium oxide, moving metallic particles of the alloy elements that have not oxidized due to the oxidation potential being greater than that of titanium or by metals ions movement by atomic diffusion from the substrate to the oxide. Regarding the removal of the oxide layer for the alloy sample after etching for 75 s, on the surface, it presents in terms of 11~12% of Mo, but on the surface, the sample of the alloy after anodization reaches 17%. Zr variation was low and remained constant between 6 and 9%. In nanotubes, it showed growth when deepening the analysis. In alloy, it is 6%, but in oxide, it is 7% (more significant than in alloy).

## 4. Conclusions

An experimental titanium alloy was processed by melting in an arc melting furnace with an argon atmosphere, melting the pure metal and hot forged by swage. The composition was fundamental to stabilize a β-titanium alloy, and equiaxial grains were 313 µm. 

On the surface of this alloy, it was possible to grow TiO_2_ nanotubes with an organic electrolyte and different anodization parameters (20 V and 30 V; 6 h and 24 h). Each combination of anodization parameters results in a different nanotube morphology. Time, voltage, and a combination of these factors played a significant role in controlling nanotube diameter, but the voltage was the factor that showed a strong influence on nanotube diameter. The highest diameter was 130 nm, and the thickness was 1294 nm; these results were obtained at parameters: 30 Volts and 24 h.

Oxide composition from Ti15Zr15Mo alloy and Ti15Zr15Mo alloy after anodization were the same elements: titanium, zirconium, molybdenum, and oxygen. However, they differ in oxygen ratio. 

There is an oxide layer in both samples. In the Ti15Zr15Mo alloy, there is a passive layer of TiO_2_. The passive layer is a thin film compared to the nanotube layer. It is possible to compare the difference in thickness by comparing the rate of oxygen in both samples; at the first layer, the rate of titanium oxygen is almost 1:2 in both samples. After the first etching (75 s), the rate of oxygen reduced above the ratio of 1:2. This reduction induced the conclusion that the layer of passive film was removed and exposed to the metal surface.

High-resolution spectra show different oxidation states of element alloys in the oxide layer. The stable form of each oxide is predominated on the surface, while the inner part is composed of suboxides and metallic forms. This results from oxygen restriction in the inner part of the oxide layer and the potential oxidation of elements.

## Figures and Tables

**Table 1:** Mean diameter and thickness of nanotube growth on the Ti15Zr15Mo anodized by 20 V and 30 V for 6 h and 24 h in an electrolyte composed of glycerol, water, and NH_2_F.

	Mean Diameter [nm]		Mean Thickness [nm]	
	20 V	30 V	20 V	30 V
6 h	75.5 ± 13	116.3 ± 32	340.0 ± 38	755.2 ± 151
24 h	77.6 ± 13	121.9 ± 35	355.5 ± 30	1294.2 ± 193

**Table 2:** XPS high resolution of Ti15Zr15Mo alloy and alloy samples after anodization in different etching times. Attribution of the peak position and relative percentage of attribution in different layers and samples.

Attribution	Position	0 s	Alloy 75 s	750 s	0 s	NTS 75 s	750 s	Citation
C 1s	[eV]	[%at]	[%at]	[%at]	[%at]	[%at]	[%at]	
C-C, C-H	285.00	66.2	66.4	65.5	79.4	76.6	85.1	[41,42]
C-O	286.16	15.3	12.9	10.2	14.7	12.9	7.9	[42,43]
C=	287.51	1.0	2.0	0.7	3.4	6.5	4.4	[43]
O-C=O	288.72	4.0	2.0	1.8	2.5	4	2.5	[44,45]
C-C, C-H	285.00	2.1	1.0	0.7	79.4	76.6	85.1	[41,42]
**O 1s**								
Oxide	530.61	45.6	24.5	21.7	58.3	40.6	40	[46,47,48,49]
TiO_2_	531.00	33.1	70.5	75.8	41	58	57.1	[50,51]
OH-	532.33	21.3	5	2.5	0.7	1.2	2.7	[52,53]
**Ti 2p3/2**								
Ti(Metallic)	453.53	4.4	65.5	68.4	0.5	2.2	1.7	[54,55,56,57,58]
Ti(II)	455.73	55.8	15.6	14.6	4.2	15.2	42.7	[56,58]
Ti(III)	457.41	19.7	8.4	8.4	1.5	34.5	21.8	[55,59,60]
TiO_2_	458.70	20	10.4	8.5	93.8	48	33.7	[57,61,62]
**Zr 3d5/2**								
Zr (Metallic)	178.90	17.3	51.6	61.7	0	0	0	[63]
ZrO	182.29	55.8	15.5	14.6	81.8	33.4	83.4	[46,64]
ZrO_2_	182.90	19.7	8.4	8.4	18.2	66.6	16.5	[59,65]
**Mo 3d5/2**								
Mo (Metallic)	226.81	32.3	41.2	31.4	0	52.9	73.6	[66,67]
MoO_2_	228.91	42.8	44.4	47.8	0	25.9	11.3	[66,68]
MoO_3_	234.32	24.9	14.4	20.7	90.8	10.9	8.7	[69,70]
Mo_2_O_5_	232.32	0	0	0	9.2	10.2	6.4	[71]

## Data Availability

Research data is available upon request.
